# Chemical and Nutritional Composition of *Terminalia ferdinandiana* (Kakadu Plum) Kernels: A Novel Nutrition Source

**DOI:** 10.3390/foods7040060

**Published:** 2018-04-12

**Authors:** Saleha Akter, Michael E. Netzel, Mary T. Fletcher, Ujang Tinggi, Yasmina Sultanbawa

**Affiliations:** 1 Queensland Alliance for Agriculture and Food Innovation (QAAFI), The University of Queensland, Health and Food Sciences Precinct, 39 Kessels Rd Coopers Plains, P.O. Box 156, Archerfield BC, QLD 4108, Australia; saleha.akter@uq.edu.au (S.A.); m.netzel@uq.edu.au (M.E.N.); mary.fletcher@uq.edu.au (M.T.F.); 2 Queensland Health Forensic and Scientific Services, Health and Food Sciences Precinct, 39 Kessels Rd, Coopers Plains, P.O. Box 594, Archerfield BC, QLD 4108, Australia; ujang.tinggi@health.qld.gov.au

**Keywords:** *Terminalia ferdinandiana*, Kakadu plum, nutrition, fatty acids, proximate, minerals, kernels

## Abstract

*Terminalia ferdinandiana* (Kakadu plum) is a native Australian fruit. Industrial processing of *T. ferdinandiana* fruits into puree generates seeds as a by-product, which are generally discarded. The aim of our present study was to process the seed to separate the kernel and determine its nutritional composition. The proximate, mineral and fatty acid compositions were analysed in this study. Kernels are composed of 35% fat, while proteins account for 32% dry weight (DW). The energy content and fiber were 2065 kJ/100 g and 21.2% DW, respectively. Furthermore, the study showed that kernels were a very rich source of minerals and trace elements, such as potassium (6693 mg/kg), calcium (5385 mg/kg), iron (61 mg/kg) and zinc (60 mg/kg) DW, and had low levels of heavy metals. The fatty acid composition of the kernels consisted of omega-6 fatty acid, linoleic acid (50.2%), monounsaturated oleic acid (29.3%) and two saturated fatty acids namely palmitic acid (12.0%) and stearic acid (7.2%). The results indicate that *T. ferdinandiana* kernels have the potential to be utilized as a novel protein source for dietary purposes and non-conventional supply of linoleic, palmitic and oleic acids.

## 1. Introduction

*Terminalia* is the second-largest genus of the combretaceae family, with approximately 250 species growing in tropical and subtropical countries around the world [1]. More than 30 species of *Terminalia* occur in northern regions of Australia [2]. More than 50 species of *Terminalia* have found utility as ingredients in foods and beverages worldwide, as preservatives, raw material for wine and palm sugar, eaten raw and as food supplements [3]. The nutritional and therapeutic properties of *Terminalia* genus can be attributed to the presence of a wide range of phytochemicals, such as phenolic compounds, which encompasses phenolic acids, gallotannins, ellagitannins, proanthocyanidins and other flavonoids [3].

*Terminalia ferdinandiana*, popularly known as Kakadu plum, is native to Australia. Indigenous Australians (Aboriginal people) have been using this plant as a food and medicine for centuries, for example, refreshing drinks are made from fresh or dried fruits in Western Australia [2]. Fruits are traditionally used as an antiseptic, soothing balm, in colds and flu and in treating a headache [4]. A number of research outcomes have been reported on the antioxidant [5,6,7], antibacterial [8,9], anti-inflammatory [10], anti-apoptotic, cytoprotective and anticancer activities [11] of *T. ferdinandiana* fruits and leaves. Phytochemical analysis has revealed that *T. ferdinandiana* fruit is a rich source of Ellagic acid and its hydrolysable tannins, ellagitannins [12]. Recently, a food safe extraction method of *T. ferdinandiana* fruits for commercial use in the food industry has been suggested [4]. Additionally, a systematic evaluation of the changes in quality and bioactivity of the fruits of *T. ferdinandiana* during processing, packaging and storage has been performed, and key chemical markers have been identified to enable standardized products to be delivered to the consumer [9].

In the last two decades, seeds and kernels from the *Terminalia* genus have been researched and reported for their nutritional properties and health-promoting activities [13,14,15]. To understand the relationship between the internal quality and genotype of the plant, studies of nut and kernels characteristics and composition are very common. During the industrial processing of *T. ferdinandiana* fruits, the seeds are treated as waste products and have been discarded. Recent studies on many fruit seeds or kernels have shown that they have the potential to be utilized as ingredients for value addition, they are very nutritious and could be used as alternate sources of essential minerals, fatty acids, and proteins [16,17,18,19].

To date, no reports have been published on the utilization of the by-products of *T. ferdinandiana* and there is no investigation on the chemical and nutritional composition of *T. ferdinandiana* kernels. The aim of this study was to determine the potential use of the by-product of *T. ferdinandiana* in the industry by determining proximate, mineral and fatty acid compositions to ascertain its nutritional value and potential as a source of food supplement ingredients for the food industry.

## 2. Materials and Methods

### 2.1. Sample Collection and Preparation

**Figure 1:**
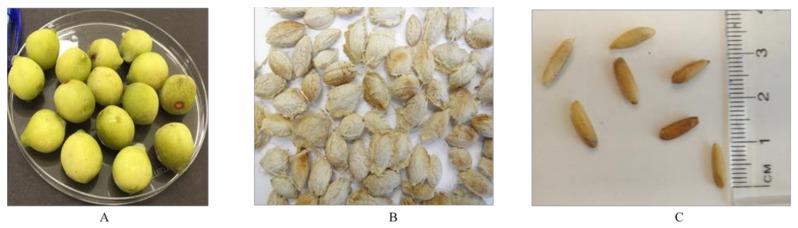
*Terminalia ferdinandiana* tissues. (**A**) Fresh fruits; (**B**) Dried seeds; (**C**) Kernels.

Fully ripe and mature fruits of *T. ferdinandiana* were collected from over 600 trees, giving a total harvest of 5000 kg, from native bush land covering an area of 20,000 km^2^ in Northern Territory, Australia in 2015 and were authenticated by the experts in Queensland Herbarium, Brisbane Botanic Gardens Mt Coot-tha, Queensland, Australia, where botanical specimens were retained for future reference (AQ522453). Seeds were collected as the by-products after pureeing of the fruits, and were stored at −20 °C prior to analysis. *T. ferdinandiana* tissues are illustrated in Figure 1.

### 2.2. Processing of Seeds

**Figure 2:**
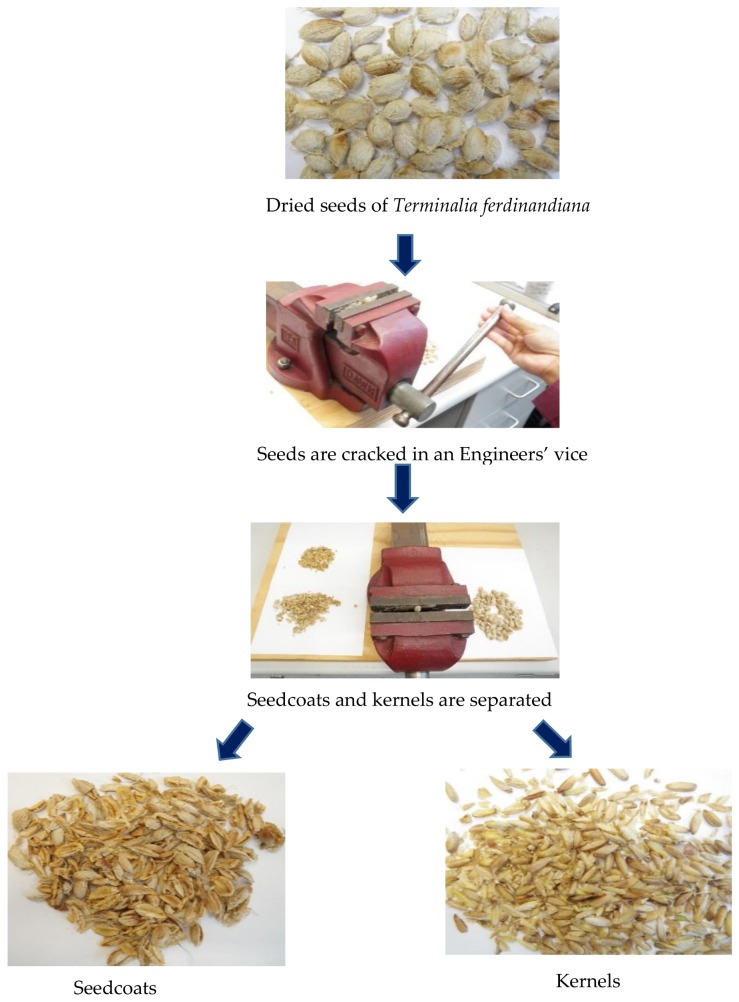
Processing of *Terminalia ferdinandiana* seeds to release kernels.

The frozen seeds were thawed, washed and cleaned manually several times to remove the pulp residues with double distilled water. The seeds were then dried in the oven for 48 h at 40 °C. After drying, the seeds were individually cracked using an Engineers’ vice size 125 (DAWN, Melbourne, Australia) to release the kernels from the seedcoats. The seedcoats and kernels were kept, processed and analyzed separately. The kernels were kept in air-tight containers and placed at −20 °C for further analysis. A flowchart depicting the processing of the seeds is illustrated in Figure 2. During the processing of fruits in the industry in a batch of 100 kg of fruits, 22 kg of seeds can be obtained as by-product. The average weight of a dry seed is 0.5 g and the moisture content is 2.8%. Average weight of a kernel is 0.04 g. The kernel is 8% of the weight of the seed. 1 kg of dry seeds can deliver 80 g of kernels. 

### 2.3. Proximate Composition Analysis

Physicochemical analysis of the kernels of *T. ferdinandiana* was performed at an accredited laboratory (National Association of Testing Authorities (NATA), Symbio Alliance, Eight Mile Plains, Queensland, Australia). The following analyses were done according to AOAC methods: vitamin C, protein (AOAC 990.03, 992.15 & 992.15), fat (AOAC 991.36), saturated, mono-unsaturated, polyunsaturated and trans-fat (AOAC 996.06), moisture (AOAC 925.10), ash (AOAC 923.03), sodium (using ICP-AES), total sugar (AOAC 977.20) and dietary fiber (AOAC 985.29, 991.42 and 993.19). Available carbohydrate and energy were calculated using FSANZ (Food Standards Australia New Zealand) codes. 

### 2.4. Fatty Acid Analysis

Dried kernels (ca. 1 g) were finely chopped and extracted with chloroform and methanol (2:1) followed by agitation at room temperature for one hour. The mixture was then centrifuged for 5 min at 3500 rpm and the whole process was repeated twice. The lipid extracts were mixed with boron trifluoride (BF_3_)-methanol reagent (20%) and fatty acids were derivatized to fatty acid methyl esters [20]. The methyl esters of the fatty acids were dissolved in heptane and analyzed by GC-MS (Shimadzu QP2010, Shimadzu Corporation, Tokyo, Japan). The GC conditions were as follows: Restek stabilwax capillary column (30 m × 0.25 mm ID × 0.5 µm film thickness) (Restek Corporation, Bellefonte, PA, USA); oven temperature program: the column held initially at 100 °C after injection and the final temperature was increased to 250 °C, total program time was 39:00 min; injector temperature: 250 °C; carrier gas: Helium; linear gas velocity: 42.7 cm/s; column flow: 1.10 mL/min; split ratio: 50.00; injection volume: 1.0 µL. MS conditions were regulated as follows: ion source temperature: 200 °C; interface temperature: 250 °C; mass range: 35–500 atomic mass units. Identification of the compounds was carried out by comparison of their retention times and mass spectra with corresponding data from a standard food industry FAME Mix (Restek Corporation, Bellefonte, PA, USA). A total of 32 individual compounds were analyzed and only the detected ones were recorded along with their quantity compared with the standard. 

### 2.5. Mineral and Trace Element Analysis

Accurately weighed 0.3 g of dried *T. ferdinandiana* kernels were taken into teflon vessels of microwave digestion system (MarsXpress, CEM, Matthews, NC, USA) and high-purity nitric acid (70% *w*/*w*, 4 mL) was added [21]. The samples were left overnight at room temperature for slow digestion gasses to evolve. The vessels were sealed and microwave-digested at increased temperature with set digestion time [22]. The digested samples were diluted and made up to 40 mL with high-purity water (Milli-Q Element system, Millipore, Bedford, MA, USA). The levels of minerals and trace elements were analyzed by inductively coupled plasma optical emission spectrometry (ICP-OES, Vista AX, Varian Australia, Mulgrave, Victoria, Australia), to measure lower levels and for greater sensitivity the analysis was carried out using ICP-MS (7500a, Agilent, Tokyo, Japan). The ICP-MS was equipped with an auto-sampler, integrated sample introduction system and a helium octopole reaction cell to remove polyatomic interferences (^40^Ag ^35^Cl on ^75^As). The operating conditions were as follows: radio frequency (RF) power 1350W, argon carrier gas 0.8 L/min and helium reaction cell gas flow rate 4.5 mL/min. The standard reference materials were used for the quality control and assurance and treated similarly to the samples throughout the study. The data of quality control and assurance are presented in the Supplementary Material (Table S1). 

### 2.6. Statistical Analysis

The data were calculated using Microsoft Excel 2013 (Microsoft Corporation, Redmond, WA, USA). The results are expressed as the mean of triplicate experiments unless otherwise specified. 

## 3. Results and Discussion

### 3.1. Proximate Composition

The proximate composition of *T. ferdinandiana* kernels is summarized in Table 1. Moisture content is an important parameter in terms of the physicochemical properties of plant parts, due to the fact that low moisture content is beneficial for retaining the quality and shelf life of seeds, and this also decreases the susceptibility for microbial growth, premature seed germination, unwarranted fermentation and undesirable biochemical changes. The moisture content is only 4% in the kernels of *T. ferdinandiana*, presenting minimum risk for microbial growth and undesirable biochemical changes upon storage. A comparable moisture content of 5.5% was reported for *T. catappa* kernels [15]. Furthermore, the results of the present study showed that the kernels were abundant in proteins, with a content of 32% relative to the standard. Protein content of *T. ferdinandiana* is higher than that of *T. catappa* kernels (20.1%) [15]. *T. sericea* kernels contain 46.2% proteins [23], which is higher than *T. ferdinandiana* kernels. Recommended dietary allowances (RDA) for protein are 56 g for a 70 kg man [24]. As the protein content of *T. ferdinandiana* kernels is high, it could be used as an alternative source or dietary supplement for consumers with restricted and compromised protein intake from other sources. Ash content is 4% and dietary fiber 21.2% in *T. ferdinandiana* kernels. Ash content signifies the presence of minerals in the kernel, tissue and the high content of fiber can help in improving the gut health and digestion. The lipid content in *T. ferdinandiana* kernels was found to be 35.1%, with less than 1% in the trans form. The WHO recommends that no more than 1% of our daily energy intake come from trans-fatty acids (TFAs). Based on the present results, it can be concluded that the fat content of *T. ferdinandiana* kernels is devoid of any trans-fat-associated health risk. The fat content in *T. sericea* seed is 32.5% [23], 64.7% in *T. catappa* kernels [15], and in *T. catappa* seed it is 32.7% [14], while in *T. ferdinandiana* kernels it is 35.1%. *T. ferdinandiana* kernels can supply 50% of the RDA of fat with saturated (SFA), monounsaturated (MUFA) and polyunsaturated (PUFA) fats are in the order of 5.8%, 9.8%, and 19.4%. These proportions are similar to the fatty acid profile determined by GC-MS (Table 2). A diet rich in PUFA is important for the structure and function of proteins, receptors, enzymes and transport molecules whereas the MUFA content may lower blood cholesterol levels, modulate immune function and can improve the fluidity of high-density lipoproteins (HDL) [25]. The results of our present study thus suggest that *T. ferdinandiana* kernels have the potential to be used as an alternative source of MUFA and PUFA.

### 3.2. Mineral and Trace Element Composition

The macro and trace element composition of *T. ferdinandiana* kernels evaluated in this study is presented in Table 3, and the non-essential and heavy metal composition is presented in Table 4. Minerals are essential for proper functioning of the body, and a deviation from the appropriate amounts can cause numerous diseases, clinical syndromes, and illnesses associated with the deficient intake, as well as overuse over time or at a certain time period of life. Hence, reference values are established and reviewed periodically to stipulate the mineral levels that will meet the needs of healthy human individuals. The RDA of the evaluated minerals for a healthy male adult of 70 kg body weight are also presented in Table 3 and Table 4. The high macro-mineral contents were found to be phosphorus 872.8 mg/100 g DW, and calcium at 538.5 mg/100 g DW, while sodium was 120.3 mg/100 g DW and magnesium 421.1 mg/100 g DW (Table 3). These results indicated that the kernels could significantly contribute to the mineral intake in humans. Mineral composition analysis of kernels from *Terminalia* genus is scarce and one report on the mineral composition of *T. catappa* seeds included phosphorus (10), calcium (36.1), magnesium (26.4), iron (375), sodium (5) and potassium (350), in mg/100 g [14]. Kernels from bayberry (*Myrica rubra*) were reported as an abundant source of potassium, containing 780 mg/100 g [26]. The potassium content of white Chinese olive (*Canarium album*) is also high, at 587 mg/100 g [19]. In our study, *T. ferdinandiana* kernels contained 669.3 mg/100 g of potassium, which can be compared to the potassium content of bayberry, Chinese olive, and black Chinese olive. Moreover, the phosphorus levels of *T. ferdinandiana* kernels (872.8 mg/100 g DW) seemed to be much higher compared to the levels of bayberry (32.9 mg/100 g) [16]. Important trace elements found in *T. ferdinandiana* were zinc, manganese, copper and iron at levels of 6, 9.1, 2.5 and 6.1 mg/100 g, respectively, and are within the RDA and AI values. It can be suggested that *T. ferdinandiana* kernels can be a valuable dietary source of these trace elements. These trace elements are important constituents of various proteins and enzymes of our body which are involved in macronutrient metabolism [15]. The levels of molybdenum, arsenic, mercury, cadmium were found at less than 0.1 mg/kg in the kernels, while the lead level was found at 0.13 mg/kg (Table 4). Heavy metal exposure poses significant health risks, which can cause life-threatening diseases, and the toxic effects are influenced by chemical forms, absorption rate, and solubility in body fluids. The toxicity of arsenic depends on the chemical form. The inorganic form of arsenic is more toxic than organic arsenic [21]. Mercury can be readily absorbed and incorporated into tissue proteins and can cause detrimental effects on health. The bioaccessibility and bioavailability of the exposed heavy metals can again vary depending on the chemical forms, time and route of exposure, duration, and concentration of the exposed metals. However, the levels of heavy metals found in *T. ferdinandiana* kernels were within the regulatory limits, suggesting that they may not impose any health risk.

### 3.3. Fatty Acid Composition

Fatty acids can be considered the main constituent of all oils and may include saturated (SFA), monounsaturated (MUFA) and polyunsaturated (PUFA) fatty acids [25]. Besides providing high-quality food, vegetable oils can also provide essential nutrients that have a particular clinical significance. PUFA are present in membrane phospholipids in some tissues and can also act as precursors for prostaglandin hormones [28]. On the other hand, SFA are reported to increase cardiovascular disease risk and sometimes can potentiate the risk of cancer and autoimmune disorders [29]. The principal fatty acid components in *T. ferdinandiana* kernels were palmitic (SFA, 12%); oleic (MUFA, 29.3%); and linoleic (PUFA, 50.2%) acids (Table 2).

SFA are reported to impact human health by increasing the plasma low-density lipoprotein (LDL) cholesterol. However, some of the SFA are also reported to increase the high-density lipoprotein (HDL) cholesterol and some of them have little or no significant role in increasing or decreasing the LDL and HDL cholesterol levels [30]. The main SFA found in *T. ferdinandiana* kernels are myristic (0.09%), palmitic (12%), stearic (7.2%), arachidic (0.76%) and behenic (0.4%) acid. The level of myristic acid in *T. ferdinandiana* kernels is only 0.09%. 

Unsaturated fatty acids can exist in *cis*- or *trans*-configuration. *Cis*-configuration is found in naturally occurring unsaturated fatty acids, while *trans*-configuration is the result of processing. *Cis*-unsaturated fatty acids are known as potent inducers of adiposomes also referred to as lipid droplets and they have important roles in cell signaling, regulation of lipid metabolism and control of the synthesis and secretion of inflammatory mediators [31]. The MUFA present in *T. ferdinandiana* kernels are palmitoleate (0.2%), oleate (29.3%) and ecosenoate (0.11%). Among the MUFA, oleic acid is the most abundant one found in *T. ferdinandiana* kernels. Oleic acid has been reported to act as an anti-inflammatory and anti-apoptotic agent. The anti-inflammatory mechanism includes down-regulating cyclooxygenase-2 and inducible nitric oxide synthase through the activation of nuclear factor-kappa B [30]. Oleic acid may promote insulin resistance which is contrary to the PUFA which protects from insulin resistance [30]. Oleic acid has also been reported to attenuate blood pressure and risk of developing hypertension [32]. The potential use of *T. ferdinandiana* kernels as a dietary source of oleic acid in reducing the risk and attenuating hypertension requires further investigation. Previous reports on some of the seeds and kernels of the family Combretaceae had reported oleic acid as the most abundant unsaturated fatty acid found in this family [33]. 

Essential PUFA are α-linolenic (18:3, *n*-3) and linoleic acid (18:2, *n*-6), from which other important PUFA are derived. Recently, essential fatty acids (EFA) have been considered as functional food components and nutraceuticals [31]. Documented roles of EFA include cardioprotective effect (due to their considerable antiatherogenic, antithrombotic, anti-inflammatory, antiarrhythmic, hypolipidemic effects), the fluidity of biological membranes, the function of membrane enzymes and receptors, modulation of eicosanoids production, blood pressure regulation and metabolism of minerals [31]. EFA are also reported to reduce the risk of cardiovascular, cancer, osteoporosis, diabetes and some other serious diseases due to their complex effects on concentrations of lipoproteins [31]. Linoleic acid is an unsaturated omega-6 fatty acid that plays a critical role in the maintenance of the structural and functional integrity of the central nervous system (CNS) and retina [23]. A deficiency can cause skin scaling and hair loss [34]. Linoleic acid (C18:2) is the only PUFA found in *T. ferdinandiana* kernels (50.2%). Therefore, it can be suggested that *T. ferdinandiana* kernels can be used as a potential dietary source of linoleic acid which can increase the systemic pool and subsequently help nourish the CNS and retina. 

The WHO recommends that total daily energy intake derived from omega-6 PUFA should be 5–8% and from omega-3 PUFA 2% for an adult male. Studies on the seeds of *T. bellirica* have reported that 40% of the seed is oil and 35% is protein and major fatty acids were linoleic (31%), palmitic (35%) and oleic (24%) acids and the authors have suggested that kernels could be used as a dietary source of linoleic acid [35]. Reported studies on various plants of *Terminalia* genus included that *T. glucausens* contains palmitic acid (34.9%), myristic acid (0.1%) and stearic acid (4.8%), seed oil of *T. superba* contains behenic acid (C22:0; 1.2%) and the oil of *T. catappa* contains stearic acid (5.8%), myristic acid (1.21%) and arachidic acid (1.3%) [33]. Variations in the fatty acid composition is very common in plants and may be due to a number of reasons including but not limited to soil composition, climate, and specific geographical locations etc. 

Nutritionally, the ratio of unsaturated to saturated fatty acids in edible oils and fats is very important. High levels of saturated fatty acids are desirable to increase oil stability. However, SFA become nutritionally undesirable, because high levels of saturated fatty acids are considered to increase the concentration of LDL, affecting the ratio of LDL to HDL and promoting vascular smooth muscle proliferation [36,37]. The ratio of UFA/SFA for *T. ferdinandiana* kernels is 4, which can be considered favorable for reducing the risk of cardiovascular complications [36]. Again, the relationship between saturated and polyunsaturated FA content is an important parameter for determination of the nutritional value of oils which is expressed as P/S index. Oils and fats with a P/S index > 1 are considered to have nutritional value. Several studies indicate that a higher P/S index means a smaller deposition of lipids in the body. The P/S indexes of *T. ferdinandiana* kernels and some other common oils and fats are shown in Table 5. The P/S index of *T. ferdinandiana* kernels was 2.45, while safflower oil is 10.55 and coconut fat is 0.005. The fatty acid composition of *T. ferdinandiana* kernels is comparable to the composition of soya bean oil (Table 5).

There are suggestions to reduce the SFA in the diet to suppress the risk of coronary heart diseases (CHD) and cardiovascular diseases (CVD). However, it is important to note that SFA reduction itself cannot suppress the risk. Mostly, reduction of SFA and TFA with their simultaneous replacement by PUFA could lead to a reduction of the risk of CHD. The SFA content of *T. ferdinandiana* kernels was 20.4%, having a very low amount of myristic acid. Based on our results, it can be concluded that the SFA of *T. ferdinandiana* kernels are unlikely to have detrimental health effects by increasing the LDL cholesterol level. Moreover, the kernels were a good source of linoleic acid suggesting *T. ferdinandiana* kernels as a valuable source of EFA that can be used in feed and food.

## 4. Conclusions

To the best of our knowledge, this is the first study on the nutritional composition of *T. ferdinandiana* kernels. The present study indicated that the kernels contain high levels of protein and lipid. The mineral composition of *T. ferdinandiana* kernels reveal a very good source of fundamental minerals and micronutrients. Furthermore, the kernels can be considered as a potential dietary source of linoleic and oleic acid. From a nutritional point of view, our present results suggest that *T. ferdinandiana* kernels have a high nutritional value and may contribute to a healthy diet. The utilization of kernels as a by-product from processing of *T. ferdinandiana* fruit will generate applications as a source of food supplement ingredients for essential fatty acids and can represent an alternate source of protein in the food and feed industry. Ongoing studies on the quality aspects of *T. ferdinandiana* kernels will also include bioaccessibility and bioavailability to substantiate the nutritional value and potential health effects of this unexploited by product.

## Figures and Tables

**Table 1:** Proximate composition of *Terminalia ferdinandiana* kernels.

			Nutrition Information (Servings per Package: 1) (Serving Size: 100 g)	
*T. ferdinandiana* Kernels			Quantity per Serving	% Daily Intake */Serving
Protein	% (*w*/*w*)	32.0	32 g	64%
Fat	% (*w*/*w*)	35.1	35.1 g	50%
Saturated Fat	% (*w*/*w*)	5.8	5.8 g	24%
Mono-unsaturated Fat	% (*w*/*w*)	9.8	9.8 g	
Poly-unsaturated Fat	% (*w*/*w*)	19.4	19.4 g	
Trans Fat	% (*w*/*w*)	<0.01	<0.1 g	
Moisture (air)	% (*w*/*w*)	4.0		
Ash	% (*w*/*w*)	4.5		
Dietary Fibre (Total)	% (*w*/*w*)	21.2	21.2 g	71%
Dry Matter	% (*w*/*w*)	96.0		
Crude Fibre	% (*w*/*w*)	11.6		
Energy	kJ/100 g	2065	24%	
Total Sugar	g/100 g	0.49	<1 g	<1%
Available Carbohydrate	%	3.2	3.2 g	1%
Sodium (Na)	mg/100 g	8.6	8.6 mg	<1%

* Percentage daily intakes are based on an average adult diet of 8700 kJ. Results are expressed as the mean of triplicate experiments.

**Table 2:** Fatty acid profile of *Terminalia ferdinandiana* kernels expressed as percentage (±SD) of the total fatty acid profile as determined by FAME GC-MS analysis.

Fatty Acid	Percentage (%) ± SD
*Saturated*	
C14:0 Methyl myristate	0.1 ± 0.01
C16:0 Methyl palmitate	12 ± 0.53
C18:0 Methyl stearate	7.2 ± 0.13
C20:0 Methyl arachidate	0.7 ± 0.06
C22:0 Methyl behenate	0.4 ± 0.13
**TSFA**	**20.4**
*Monounsaturated*	
C16:1 (*cis*-9) Methyl palmitoleate	0.1 ± 0.06
C18:1 (*cis*-9) Methyl oleate	29.2 ± 0.68
C20:1 (*cis*-11) Methyl eicosenoate	0.1 ± 0.04
**TMUFA**	**29.4**
*Polyunsaturated*	
C18:2 (all-*cis*-9,12) Methyl linoleate	50.2 ± 1.1
**PUFA**	**50.2**
SFA vs. UFA	0.25:1
MUFA vs. PUFA	0.6:1

SFA: saturated fatty acids; UFA: unsaturated fatty acids; MUFA: monounsaturated fatty acids; PUFA: polyunsaturated fatty acids; TSFA: total saturated fatty acids; TMUFA: total monounsaturated fatty acids; TPUFA: total polyunsaturated fatty acids; data presented as a mean ± SD of triplicate experiments.

**Table 3:** Major and trace elements composition of *Terminalia ferdinandiana* kernels (mg/100 g DW).

Mineral Composition															
Major Elements						Micro/Trace Elements									
	Ca	Mg	Na	K	P	Fe	Zn	Mn	Cu	Co	Ni	Mo	Se	Sr	B
Kernels (mg/100 g DW)	538.5	421.1	120.3	669.3	872.8	6.1	6.0	9.1	2.5	0.02	0.17	<0.01	0.02	5.0	0.83
DRI	1200 AI ^a^	350 EAR ^a^	1.3 AI ^a^	4.7 AI ^a^	700 RDA ^a^	8 RDA ^a^	11 RDA ^a^	2.3 AI ^a^	700 RDA ^a^	0.12AI ^a^	1.0 UL ^a^	34 EAR ^a^	45 EAR ^a^	1–5 RDA ^a^	20 UL ^a^
Units	mg	mg	g	g	mg	mg	mg	mg	µg	µg	mg	µg	µg	mg	mg

Results are expressed as the mean of duplicate experiments. RDA—recommended dietary allowance, AI—adequate Intake, UL—tolerable upper intake level, DRI—Dietary reference intakes; EAR—Estimated average requirement. ^a^ Institute of Medicine. 2006. *Dietary Reference Intakes*: *The Essential Guide to Nutrient Requirements*. Washington, DC: The national academic press. ISBN: 978-0-309-15742-1. doi:10.17226/11537. URL https://www.nap.edu/read/11537/chapter/1. Accessed on 17 February 2017.

**Table 4:** Non-essential elements and heavy metal compositions of *Terminalia ferdinandiana* kernels (mg/100 g DW).

	Non-Essential Elements	Heavy Metals			
	Ba	As	Hg	Pb	Cd
Kernels (mg/100 g DW)	0.31	<0.01	<0.01	0.013	<0.01
DRI	0.02 UL ^a^	12.5–25 UL ^b^	5 UL ^c^	25 UL ^c^	2.5 UL ^d^
Units	mg/kg BW	µg/kg BW/week	µg/kg BW/week	µg/kg BW/week	µg/kg BW/week

Results are expressed as the mean of duplicate experiments. UL—tolerable upper intake level, DRI—Dietary reference intakes; BW—body weight; ^a^ Scientific Committee on Health and Environmental Risks (2012). Assessment of the tolerable daily intake of barium. European commission. URL http://ec.europa.eu/health/scientific_committees/environmental_risks/docs/scher_o_161.pdf. Accessed 17 February 2017; ^b^ Food Safety authority of Ireland (2009). Mercury, Lead, Cadmium, Tin, and Arsenic in Food. Toxicology factsheet series, Issue no. 1. URL www.fsai.ie/WorkArea/DownloadAsset.aspx?id=8412. Accessed 17 February 2017; ^c^ [27]; ^d^ Statements on the tolerable weekly intake for cadmium. Panel on contaminants in the food chain. EFSA, 2011, 9(2). URL https://www.efsa.europa.eu/en/efsajournal/pub/1975. Accessed on 17 February 2017.

**Table 5:** Comparison of the fatty acid compositions of *Terminalia ferdinandiana* kernels with commonly consumed oils and fats.

Type of Oil/Fat	Fatty Acid Composition			P/S Index	Reference
	SFA	MUFA	PUFA		
*T. ferdinandiana* Kernels	20.4	29.6	50.0	2.45	Current study
Coconut	90.5	8.8	0.5	0.005	[36]
Corn	25.1	26.8	48	1.91	
Cottonseed	22.4	35.4	42	1.87	
Soybean	13.5	28.5	57.5	4.26	
Peanut	19.2	58.5	20	1.04	
Safflower	7.2	16.6	76	10.55	
Linseed	9.65	22.1	68	7.05	
Palm kernel	76	22.5	1.25	0.016	
Sunflower seed	8.8	31.5	59.5	6.76	
Canola	9.6	59.5	30.7	3.2	

## Supplementary Materials

The following are available online at http://www.mdpi.com/2304-8158/7/4/60/s1, Table S1: Trace element recoveries from reference materials.
