# Supplementary material for: Chemical and Nutritional Composition of Terminalia ferdinandiana (Kakadu Plum) Kernels: A Novel Nutrition Source
Source: Foods. 2018 Apr 12;7(4):60. doi: 10.3390/foods7040060 (PMC5920425; doi:10.3390/foods7040060)
Supplement: Supplementary file 1 [file foods-07-00060-s001.pdf]

Table S1: Supplementary Material (Trace element recoveries from reference materials)

Reference Materials	B mg/kg	Na mg/kg	Mg mg/kg	K mg/kg	Ca mg/kg	Mn mg/kg	Fe mg/kg	Co mg/kg	Ni mg/kg	Cu mg/kg	Zn mg/kg
SRM 1570 Spinach											
This study											
Mean (n=7)[a]	29	13915	8092	34271	11891	161	442	1.3	4.7	11.9	47.01
s.d.[b]	1.3	363	162	504	272	2.8	29.7	0.02	0.14	0.14	0.69
Reference value	30	-	-	35600	13500	165	550	1.5	6.0	12	50
s.d.	-	-	-	300	300	6	20	-	-	2	2
Recovery (%)[c]	95	-	-	96	88	98	80	89	78	99	94
IRMM-804 Rice flour											
This study											
Mean (n=4)	2.05	-	1517	3752	108	34	9.93	-	0.22	2.8	21.7
s.d.	0.68	-	20	207	2.7	0.87	0.59	-	0.01	0.03	0.48
Reference vaue	-	-	-	-	-	34.2	-	-	-	2.74	23.1
s.d.	-	-	-	-	-	2.3	-	-	-	0.24	1.9
Recovery (%)	-	-	-	-	-	99	-	-	-	101	94
DC73350 Poplar leaves											
This study											
Mean (n=4)	59	-	6517	14971	18039	44	207	0.45	1.72	8.88	36.11
s.d.	1.38	-	167	880	212	0.28	21.60	0.01	0.02	0.04	0.30
Reference value	-	-	-	13800	18100	45	247	0.42	1.9	9.3	37
s.d.	-	-	-	700	1300	4	17	0.03	0.3	1.0	3
Recovery (%)	-	-	-	108	100	99	84	107	90	95	98

[a]n=number of replicate analyses

[b]s.d. = standard deviation of the mean

[c]Recovery (%) is calculated as: (determined mean/reference mean) x 100

As<br>mg/kg	Se<br>mg/kg	Sr<br>mg/kg	Mo<br>mg/kg	Cd<br>mg/kg	Ba<br>mg/kg	Hg<br>mg/kg	Pb<br>mg/kg
0.15	0.18	74.53	0.27	1.37	10.13	0.03	0.96
0.01	0.04	1.83	0.03	0.01	0.17	0.00	0.04
0.15	-	87	-	1.5	-	0.030	1.2
0.05	-	2	-		-	0.005	0.2
101	-	86	-	91	-	94	80
0.05	0.12	0.16	0.41	1.50	0.20	-	0.39
0.00	0.03	0.01	0.01	0.01	0.005	-	0.01
0.049	-	-	-	1.61	-	-	0.42
0.004	-	-	-	0.07	-	-	0.07
106	-	-	-	93	-	-	94
0.34	0.27	141.31	0.19	0.37	24.62	-	1.37
0.01	0.02	0.53	0.00	0.01	0.23	-	0.02
0.37	0.14	154	0.18	0.32	26	-	1.5
0.09	0.02	9	0.01	0.07	4	-	0.3
92	193	92	104	116	95	-	91
